# Distal Tibia Apex Posterior Angulation: A Normal Anatomic Variant Related to Hindfoot Alignment With Side-to-Side Symmetry

**DOI:** 10.5435/JAAOSGlobal-D-23-00076

**Published:** 2023-06-15

**Authors:** Mark F. Megerian, Gregory V. Schimizzi, Kathryn A. Barth, Scott M. LaValva, Craig E. Klinger, Daniel R. Dziadosz, William M. Ricci, Sean T. Campbell

**Affiliations:** From the Orthopaedic Trauma Service, Hospital for Special Surgery, New York, NY (Megerian, Schimizzi, Dr. Barth, Dr. LaValva, Klinger, Dr. Dziadosz, and Dr. Ricci); the Case Western Reserve University School of Medicine, Cleveland, OH (Megerian); and the Department of Orthopaedic Surgery, UC Davis Medical Center, Sacramento, CA (Dr. Campbell).

## Abstract

**Objectives:**

The sagittal plane of the distal tibia has not been well-described. This study sought to characterize sagittal plane morphology, determine symmetry from side to side, and identify differences based on hindfoot alignment.

**Methods:**

One hundred twelve bilateral lateral weight-bearing ankle radiographs were retrospectively evaluated (224 ankles). Hindfoot alignment was classified as neutral, planus, or cavus using the Meary angle. The angle between the diaphyseal and distal tibia axes was measured, and the apex location relative to the plafond was recorded.

**Results:**

A mean distal tibia apex posterior angulation (DTAPA) of 2.0° (range −2° to 7°, SD = 2.06°) was located 8.0 cm proximal to the plafond. No difference was observed from side to side in DTAPA magnitude (*P* = 0.36) or location (*P* = 0.90). Planus alignment was associated with a significantly greater DTAPA (3.05°) as compared with neutral (1.89°) (*P* = 0.002) and cavus (1.25°) (*P* < 0.001) alignment.

**Conclusion:**

The distal tibia has an apex posterior angulation, suggesting that the true anatomic axis of the tibia terminates just posterior to the plafond center. Hindfoot alignment is related to distal tibia morphology. DTAPA symmetry indicates that contralateral imaging can be used to guide reconstruction of patient-specific anatomy and alignment. Knowledge of the DTAPA may help mitigate sagittal malalignment during distal tibia fracture surgery.

Medullary nailing is a common treatment for fixation of tibia fractures; however, higher rates of malalignment have been reported when compared with plate fixation in the setting of proximal and distal 1/3 fractures.^1,2,3,4,5^ Obtaining and maintaining anatomic alignment during fracture reduction requires an understanding of the normal distal tibia morphology in both the coronal and sagittal planes. Studies have shown that in the coronal plane, the true anatomic tibial axis intersects the plafond laterally rather than centrally and that ideally, a medullary implant should terminate in the lateral portion of the distal segment.^6-8^ This coronal anatomy helps explain the increased incidence of valgus malalignment of distal tibia fractures treated with medullary implants that terminate medial of center.^5,9,10^

In the sagittal plane, it has been previously demonstrated that anterior distal nail targets are associated with relative procurvatum and a malalignment rate three times that of central or posterior nail targets.^5^ In fact, a central or slightly posterolateral nail target was independently associated with a 96% decrease in the odds of malalignment.^5^ Similar to the coronal plane, there may be an anatomical variant in the sagittal plane of the distal tibia that might explain the risk of malalignment associated with anterior nail placements. However, the native sagittal plane anatomy of the distal tibia metaphysis relative to that of the proximal diaphysis has never been studied. Characterization of this osseous anatomy may be helpful to achieve adequate sagittal alignment during distal tibia fracture surgery. Furthermore, knowledge that the contralateral distal tibia is symmetrical to the sagittal plane would offer the surgeon a reliable template to achieve accurate reduction in cases of complex distal tibia and pilon fractures.

Therefore, the primary purpose of this study was to investigate the native sagittal plane anatomy of the distal tibia and determine whether side-to-side symmetry exists. Secondarily, we sought to define other ankle radiographic parameters that may aid in distal tibia fracture reduction, including sagittal plane talar station, anterior distal tibia angle, and plafond radius of curvature, as well as investigate the influence of distal tibia morphology on hindfoot alignment. We hypothesized that the distal tibia has an apex posterior angulation that is symmetric from side to side and is correlated with hindfoot alignment.

## Methods

### Inclusion and Exclusion Criteria

Bilateral lateral weight-bearing ankle radiographs of skeletally mature patients without osseous pathology from a single academic orthopaedic specialty hospital between January 2020 and December 2021 were retrospectively evaluated after obtaining institutional review board approval. Exclusion criteria were (1) age younger than 18 years, (2) previous or current osseous injury, (3) presence of surgical implants, (4) lack of a calibration marker, and (5) inadequate lateral imaging, with adequacy defined as concentric overlap of the talar dome, visualization of the tibial diaphysis and a minimum of 150 mm proximal to the plafond, and inclusion of the metatarsals. Indications for imaging were evaluated to ensure compliance with inclusion criteria. In total, 201 patients with bilateral lateral weight-bearing ankle radiographs were identified and evaluated. After exclusion, 112 patients were included in the retrospective review for a total of 224 ankles. The mean patient age was 46 (range 18 to 79) years. There were 61 female (54%) and 51 male (46%) patients. Acute pain was the most frequent indication, most commonly about the ankle (70.5%), followed by Achilles tendon (10.7%), foot (0.9%), and calf (0.9%) pain. Chronic pathology included ankle pain secondary to chronic sprains (8.9%), flatfoot deformity (2.7%), and inflammatory arthritis (0.9%). Five healthy athletes (4.5%) required imaging as per annual team or predraft physical examinations.

### Measurements

Radiographic measurements were made using a four-point angle function and a virtual ruler rounded to the nearest 0.1 mm on a universally licensed image archiving and communications system (Sectra, IDS7, Sectra AB, Sweden). Every radiograph was calibrated using a calibration marker to standardize measurements and ensure accuracy. All measurements were made by two independent investigators.

**Figure 1:**
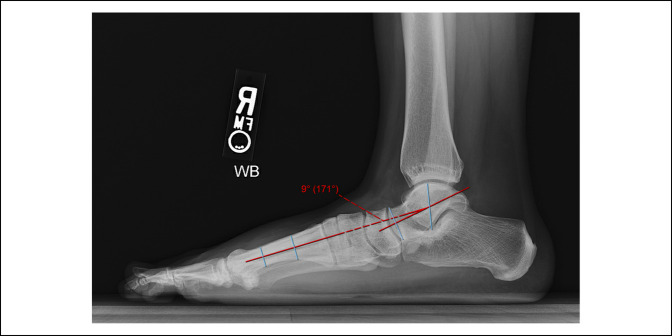
Radiograph showing an example of the Meary angle (talo-first metatarsal angle), an angle measured between a line bisecting the talar body, neck, and head and a line through the long axis of the first metatarsal diaphysis. This example demonstrates pes planus hindfoot alignment with a Meary angle of 9°.

Hindfoot alignment was categorized using the Meary angle (talo-first metatarsal angle), an angle measured between a line bisecting the talar body, neck, and head and a line through the long axis of the first metatarsal diaphysis (Figure 1). A Meary angle <4° is considered neutral, whereas an angle ≥4° is considered abnormal with varying degrees of severity.^11^ Mild abnormalities are between 4° and 15°; moderate abnormalities are between 15° and 30°; and severe abnormalities are greater than 30°.^11^ An abnormal Meary angle with a downward apex is considered pes planus and that with an upward apex is considered pes cavus. This is a previously described classification schema for hindfoot alignment based on Meary angle^11^ and the current system used by our institutions. For purposes of this study, angles with a downward apex were assigned negative values and angles with an upward apex were assigned positive values. Therefore, hindfoot alignment was categorized as neutral for any Meary angle between −4° (apex down) and 4° (apex up), pes planus for any Meary angle less than −4° (apex down), and pes cavus for any Mearys angle greater than 4° (apex up).

**Figure 2:**
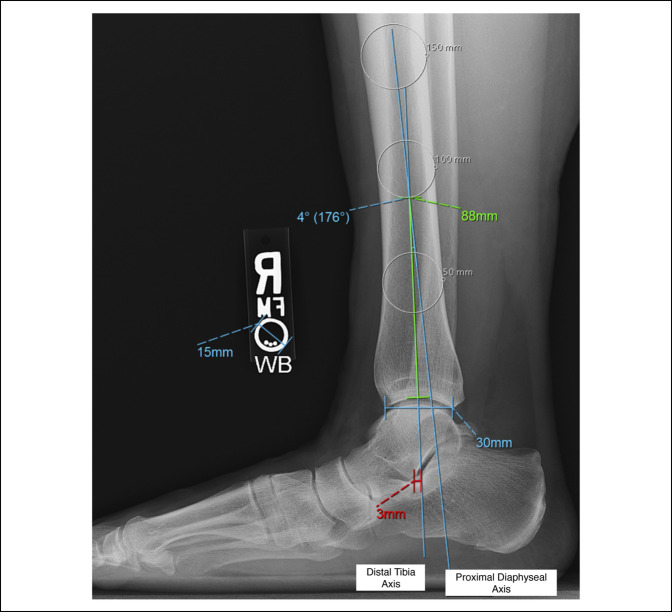
Radiograph showing an example demonstrating a distal tibia apex posterior angulation of 4° located 88 mm proximal to the plafond. The lateral talar process was located 3 mm anterior to the distal tibia axis. The proximal diaphyseal axis was defined by a line connecting two points 150 and 100 mm proximal to the plafond. The distal tibia axis was defined as a line connecting a point 50 mm proximal to the plafond to the bisection of a line connecting the anterior and posterior lips of the plafond. All described points at each respective level were equidistant from the anterior and posterior outer cortices. The location of the CORA relative to the plafond was measured along the distal tibia axis. The distal tibia axis was then extended to a point below the lateral talar process, such that the horizontal distance (using a line parallel to the floor) from the lateral talar process to the distal tibia axis could be measured. The 15-mm marker indicates that the radiograph was properly calibrated to ensure accurate measurements. CORA = center of rotation of angulation

**Figure 3:**
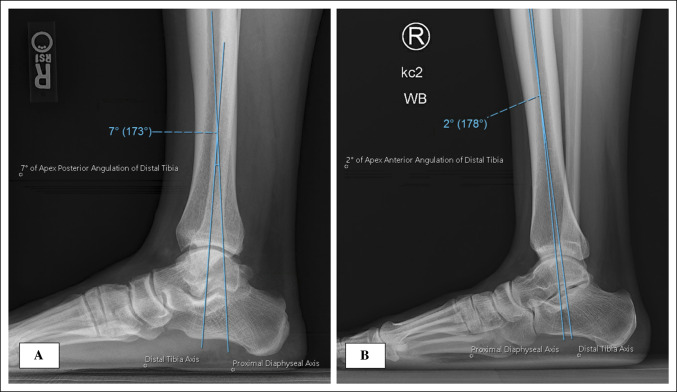
Radiographs showing an example demonstrating the measurement of distal tibia angulation. **A**, Distal tibia apex posterior angulation of 7°. Note that the proximal diaphyseal axis is posterior to the distal tibia axis. **B**, Distal tibia apex anterior angulation of 2°. Note that the proximal diaphyseal axis is anterior to the distal tibia axis.

Distal tibia morphology was assessed by measuring the angle between the proximal diaphyseal and distal tibia axes (Figure 2). The proximal diaphyseal axis was defined by a line connecting two points 150 and 100 mm proximal to the plafond (Figure 2). These points were the most consistent distances proximal to the plafond across lateral weight-bearing ankle radiographs that provided satisfactory visualization of the tibial diaphysis to gauge the proximal anatomic axis. The distal tibia axis was defined as a line connecting a point 50 mm proximal to the plafond to the bisection of a line connecting the anterior and posterior lips of the plafond (Figure 2). Each point used to define the axes was equidistant from the anterior and posterior outer cortices of the tibia. The center of rotation of angulation (CORA) was defined as the intersection point between the proximal diaphyseal and distal tibia axes, and the angle value was recorded. Angles with a posterior apex intersection point (ie, proximal diaphyseal axis posterior to the distal tibia axis) were considered “apex posterior” and assigned positive values, whereas angles with an anterior apex intersection point (ie, proximal diaphyseal axis anterior to the distal tibia axis) were considered “apex anterior” and assigned negative values. See Figure 3 for an example of both apex posterior (Figure 3, A) and apex anterior (Figure 3, B) angulations. The location of the CORA relative to the plafond was measured along the distal tibia axis. The distal tibia axis (Figure 2) was then extended to a point below the lateral talar process such that the horizontal distance (using a line parallel to the floor) from the lateral talar process to the distal tibia axis could be measured (Figure 2). Positive values indicated anterior positioning of the lateral talar process relative to the distal tibia axis, whereas negative values indicated posterior positioning.

**Figure 4:**
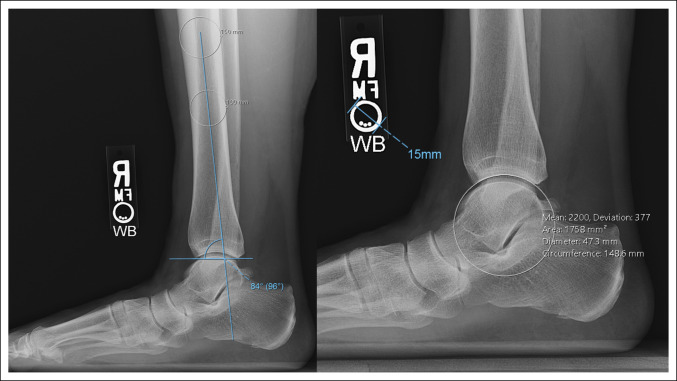
Radiographs showing an example demonstrating measurements of the anterior distal tibial angle (left) and the plafond radius of curvature (right).

The anterior distal tibia angle was measured between the proximal diaphyseal axis and a line connecting the anterior and posterior lips of the tibial plafond. The plafond radius of curvature (ROC) was determined by fitting a circle with the same tangent as the plafond articular surface (Figure 4). Using the area of this circle, the image archiving and communications system back-calculated the circle radius, which was deemed as the plafond ROC.

### Statistical Analysis

Standard descriptive statistics (eg, percentage rates for categorical variables; mean, range, and SD [σ] for continuous variables) were used to summarize demographic and radiographic data. Normality of distribution of continuous variables was assessed using a Kolmogorov-Smirnov test. Depending on the normality, radiographic parameters were compared between bilateral ankles and sexes using a paired Student *t*-test or a Mann-Whitney *U* test. Continuous variables related to hindfoot alignment (pes planus, neutral, and pes cavus) were compared using a Kruskal-Wallis one-way analysis of variance test. Correlations between continuous variables were assessed using a linear regression model and quantified with a Pearson correlation coefficient (r) and coefficient of determination (R^2^). Two authors reviewed the bilateral radiographs of 20 patients for Meary angle, distal tibia morphology, CORA location, lateral talar process relative location, anterior distal tibia angle, and plafond ROC, totaling 40 unique measurements per variable. Measurements were made 4 months apart to determine interobserver and intraobserver reliability. All statistical analyses were conducted using Statistical Package for the Social Sciences (Version 28.0.1.0).

## Results

Using the Meary angle, 56 feet (25%) were classified as pes planus, 106 (47%) were classified as neutral, and 62 (28%) were classified as pes cavus. No significant difference was observed in the Meary angle between right and left feet (*P* = 0.17), indicating bilateral correlation of hindfoot alignment. Laterality and sex comparisons are summarized for all radiographic parameters in Table 1 and Table 2, respectively.

**Table 1:** Comparisons by Laterality

	Overall Mean (n = 224)	Right (n = 112)	Left (n = 112)	*P*
Distal tibia angulation	2.0°, apex posterior	1.9°, apex posterior	2.1°, apex posterior	0.36
CORA location (cm proximal to the plafond)	8.0 cm^a^	8.7 cm^b^	7.9 cm^c^	0.90
Lateral talar process to the distal tibia axis	5.9 mm	5.7 mm	6.1 mm	0.46
Anterior distal tibial angle	83.6°	83.6°	83.6°	0.97
Plafond radius of curvature	22.9 mm	22.9 mm	23.0 mm	0.93
Meary angle	−0.3° (apex down)	0.3° (apex up)	−1.0° (apex down)	0.17

CORA = center of rotation of angulation

Please note that because not all patients necessarily had an angulation in the distal tibia (either apex posterior or apex anterior), that not all patients, therefore, had a corresponding “CORA location.” Thus, the n values for CORA location differ for the overall, right, and left radiographs.

a n = 180 for the overall cohort.

b n = 88 for right radiographs.

c n = 92 for left radiographs.

**Table 2:** Comparisons by Sex

	Overall Mean (n = 224)	Men (n = 102)	Women (n = 122)	*P*
Distal tibia angulation	2.0°, apex posterior	2.8°, apex posterior	1.4°, apex posterior	**<0.001**
CORA location (cm proximal to the plafond)	8.0 cm^a^	8.1 cm^b^	8.0 cm^c^	0.72
Lateral talar process to the distal tibia axis	5.9 mm	6.5 mm	5.4 mm	**0.005**
Anterior distal tibial angle	83.6°	82.2°	84.9°	**<0.001**
Plafond radius of curvature	22.9 mm	24.5 mm	21.7 mm	**<0 0.001**
Meary angle	−0.3°, apex down	−0.84°, apex down	0.04°, apex up	0.77

CORA = center of rotation of angulation

Please note that because not all patients necessarily had an angulation in the distal tibia (either apex posterior or apex anterior), that not all patients, therefore, had a corresponding “CORA location.” Thus, the n values for CORA location differ for the overall cohort, men, and women.

a n = 180 for the overall cohort.

b n = 86 for men.

c n = 94 for women.

### Distal Tibia Sagittal Plane Angulation

**Figure 5:**
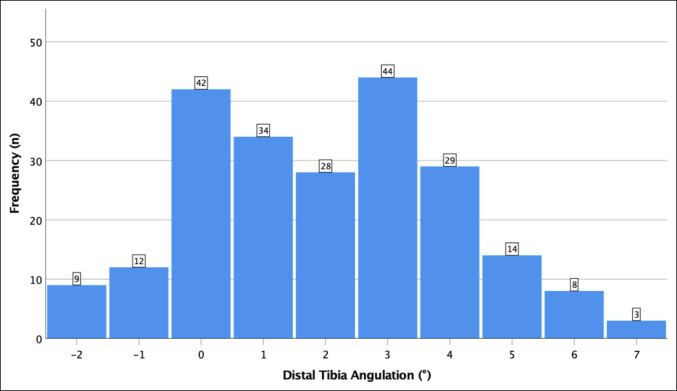
Graph showing the overall distribution of distal tibia angulation values. Positive values indicate an apex posterior angulation, and negative values indicate an apex anterior angulation. Average 2° apex posterior, range 2° apex anterior to 7° apex posterior, σ = 2.06°.

**Figure 6:**
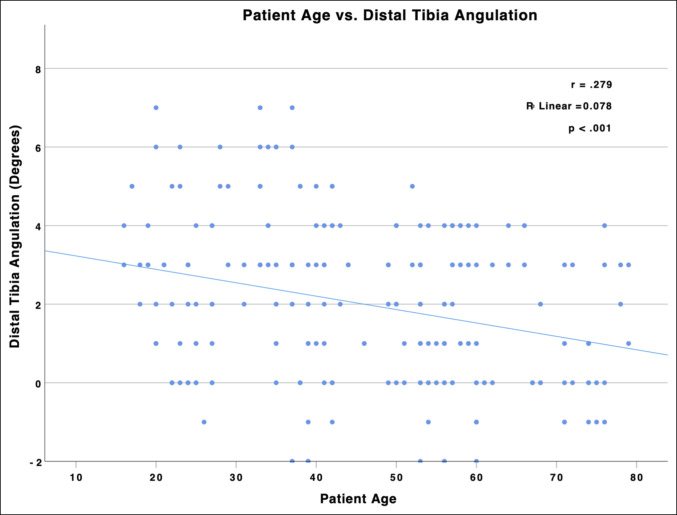
Graph showing patient age plotted against distal tibia angulation. Linear regression revealed that the DTAPA negatively correlated with patient age, demonstrating a lesser magnitude apex posterior angulation in older patients (r = 0.279, R2 = 0.078, *P* < 0.001). DTAPA = distal tibia apex posterior angulation

The distribution of distal tibia angulation in the sagittal plane is shown in Figure 5. Overall, there was a mean distal tibia apex posterior angulation (DTAPA) of 2.0° (range −2° to 7°, σ = 2.06°) with the CORA located 8.0 cm proximal to the plafond (range −8.1 to 30.5 cm, σ = 4.2 cm). Of the 224 ankles evaluated, only 21 ankles (9.4%) exhibited an apex anterior angulation. See Figure 3 for an example of both apex posterior (Figure 3, A) and apex anterior (Figure 3, B) angulations. No difference was noted in magnitude of the angulation (*P* = 0.36) or distance from the plafond to the CORA (*P* = 0.90) by laterality, demonstrating side-to-side symmetry of distal tibia morphology. The DTAPA was significantly larger in men (2.8°) than in women (1.4°) (*P* < 0.001), with no difference in CORA location between sexes (*P* = 0.72). The DTAPA negatively correlated with patient age, demonstrating a lesser magnitude apex posterior angulation in older patients (r = 0.279, R2 = 0.078, *P* < 0.001) (Figure 6).

### Ankle Radiographic Parameters

Relative to the distal tibia axis, the lateral process of the talus was located anterior in 95.6% of ankles, central (ie, directly in line) in 3.6%, and posterior in 0.8%. On average, the lateral talar process was located 5.9 mm anterior to the distal tibia axis (range −3 to 14 mm, σ = 2.7 mm). No difference was noted in lateral talar process location by laterality (*P* = 0.46), but it was more anterior in men (6.5 mm) than in women (5.4 mm) (*P* = 0.005).

The average anterior distal tibia angle was 83.6° (range 74° to 96°, σ = 3.3°). No difference was observed in anterior distal tibia angle by laterality (*P* = 0.97), but the magnitude of angulation was significantly greater in women (84.9°) than in men (82.2°) (*P* < 0.001).

The average plafond ROC was 22.9 mm (range 16 to 32 mm, σ = 3.0). No difference was noted in plafond ROC by laterality (*P* = 0.93), but the magnitude of the plafond ROC was significantly greater in men (24.5 mm) than in women (21.7 mm) (*P* < 0.001).

### Association With Hindfoot Alignment

**Figure 7:**
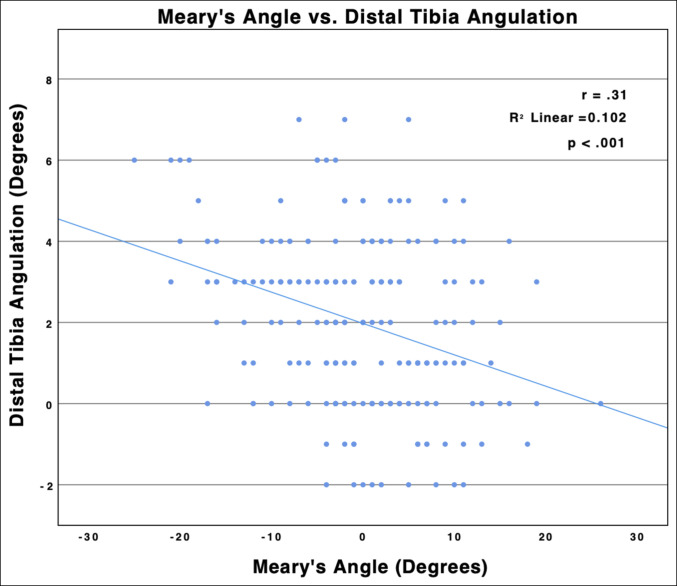
Graph showing Meary angle plotted against distal tibia angulation. Negative Meary angles reflect more pes planus alignment (apex down), and positive Meary angles reflect more pes cavus alignment (apex up). Linear regression revealed a correlation between the distal tibia apex posterior angulation (DTAPA) and the Meary angle (r = 0.32; R2 = 0.102; *P* < 0.001), with the DTAPA increasing as the Meary angle transitioned from pes cavus to pes planus hindfoot alignment.

**Figure 8:**
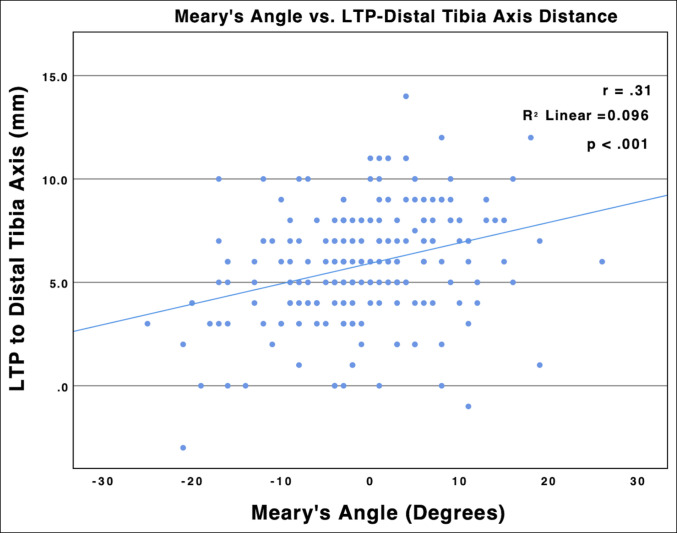
Graph showing Meary angle plotted against lateral talar process (LTP)–to–distal tibia axis distance. Negative Meary angles reflect more pes planus alignment (apex down), and positive Meary angles reflect more pes cavus alignment (apex up). Linear regression revealed a correlation between the relative lateral talar process location and the Meary angle (r = 0.31; R2 = 0.096; *P* < 0.001), with the lateral talar process shifting further anterior from the distal tibia axis as the Meary angle transitioned from pes planus to pes cavus hindfoot alignment.

A statistically significant correlation was found between the DTAPA and the Meary angle (r = 0.32; R2 = 0.102; *P* < 0.001), with increasing sagittal plane apex posterior angulation as foot alignment transitioned from pes cavus to planus (Figure 7). On average, planus alignment was associated with a significantly greater DTAPA (3.05°) as compared with neutral (1.89°) (*P* = 0.002) and pes cavus (1.25°) (*P* < 0.001) alignments. A statistically significant correlation was found between the lateral talar process location and the Meary angle (r = 0.31; R2 = 0.096; *P* < 0.001) (Figure 8), with the lateral talar process significantly more anterior in pes cavus (6.70 mm) than pes planus (4.77 mm) (*P* < 0.001) morphology. No significant differences were observed in relative CORA location (*P* = 0.99), anterior distal tibia angle (*P* = 0.94), or plafond ROC (*P* = 0.27) between hindfoot alignment groups.

### Measurement Reliability

Intraobserver reliability was found to be excellent for Meary angle (ICC = 0.95), anterior distal tibia angle (ICC = 0.98), plafond ROC (Intraclass correlation coefficient [ICC] = 0.96), DTAPA (ICC = 0.97), and lateral talar process relative location (ICC = 0.96) and good for CORA location (ICC = 0.88). Interobserver reliability was found to be excellent for Meary angle (ICC = 0.93), anterior distal tibia angle (ICC = 0.97), plafond ROC (ICC = 0.95), DTAPA (ICC = 0.92), and lateral talar process relative location (ICC = 0.91) and acceptable for CORA location (ICC = 0.73).

## Discussion

In this radiographic study, we identified consistent sagittal plane DTAPA that was symmetric from side to side. A correlation was found between the magnitude of this angulation and increasing pes planus foot alignment. In addition, cavus alignment was correlated with anterior translation of the lateral process of the talus relative to the distal tibia sagittal axis.

Historically, a “center-center” end point in the coronal and sagittal planes was thought to be desirable during tibial nailing.^10,12^ More recently, in a CT analysis of 860 tibiae, Trompeter et al^6^ found the ideal tibial nail path to be 4.4 mm lateral to the center of the plafond. Aneja et al^7^ and Schumaier et al^8^ similarly described a point 60% to 64% of the medial-lateral plafond width from the medial tibial cortex. These studies and others have described a relationship between a lateral nail end point and a decreased rate of valgus malalignment of the tibia.^5,9,10^ The ideal end point in the sagittal plane is less robustly studied. In a review of 130 distal tibia fractures treated with intramedullary nailing, Brinkman et al^5^ found that the rate of sagittal malalignment was 3.3 times more likely for nails directed anterior (19.2%) as compared with central or posterior nails (5.8%). Relative procurvatum was similarly exacerbated with anterior nails (mean ADTA 82.8° versus 80.9°, *P* < 0.01).^5^ Most notably, multivariate analysis determined that a central or slightly posterolateral nail target was independently associated with a 96% decrease in odds of malalignment.^5^ In a similar review of 203 distal tibia fractures, Triantafillou et al found a 100% (4/4) rate of sagittal malalignment when the distal nail tip was placed in the far anterior quadrant, 50% (22/44) when placed in the anterior middle quadrant, and 31.3% (5/16) when placed in the posterior middle quadrant. Notably, no nails were placed in the far posterior quadrant.^9^ Both studies concluded that in the sagittal plane, nails should be targeted centrally or posteriorly to mitigate risk of sagittal malalignment.^5,9^ Brinkman et al^5^ further suggested targeting nails slightly posterolaterally to as well account for coronal anatomy. Both described studies were observational, and neither investigated a causal explanation for their findings.

In consideration of the clear influence of distal nail position on sagittal alignment, the existence of the DTAPA may offer an anatomic explanation for the reduced incidence of sagittal malalignment with posterior nail targets. Our findings related to the DTAPA may suggest that the true anatomic axis of the tibia lies just posterior of the plafond center. It is important to underscore that our study was purely a radiographic study, and we did not evaluate the influence of distal nail tip disposition on sagittal alignment. Thus, we can only extrapolate our findings to help contextualize conclusions from prior literature. Future investigations of distal nail placement for distal tibia fractures should assess for the influence of the DTAPA on sagittal alignment. In addition, given the increased DTAPA magnitude in those with pes planus hindfoot alignment, it would be interesting to assess malalignment rates in those with pes planus versus neutral and pes cavus hindfoot alignment.

**Figure 9:**
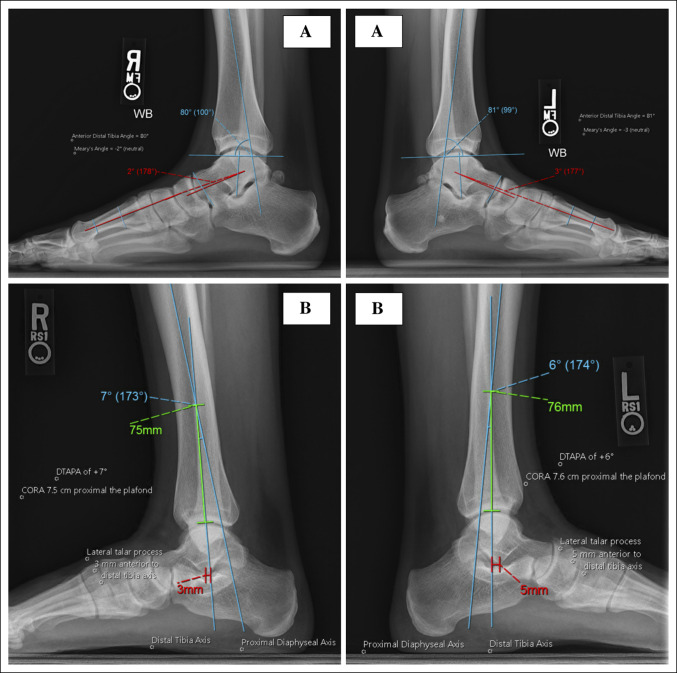
Radiographs showing radiographic parameters depicted on bilateral imaging. **A**, Bilateral imaging from one patient showing the anterior distal tibia angle (right: 80°, left: 81°) and Meary angle (right: 2° [apex down], left: 3° [apex down]). **B**, Bilateral imaging from another patient showing the DTAPA (right: 7°, left: 6°), CORA location proximal to the plafond (right: 7.5 cm, left: 7.6 cm), and lateral talar process location relative to the distal tibia axis (right: 3 mm anterior, left: 5 mm anterior). CORA = center of rotation of angulation, DTAPA = distal tibia apex posterior angulation

Although a consistent DTAPA exists proximal to the plafond, there is considerable variation from one patient to another. The DTAPA was markedly greater in male patients, younger patients, and in those with pes planus hindfoot alignment. However, there was no statistical difference in degree and location of the DTAPA from side to side. Our findings suggest that imaging of the uninjured contralateral distal tibia can be reliably used as a template for normal anatomy to help restore patient-specific sagittal alignment. Furthermore, we as well found no difference from side to side for the anterior distal tibia angle and plafond ROC, results that corroborate findings from Kellam et al,^13^ and no difference in the talus position, a novel finding. See Figure 9 for depiction of bilateral correlation between these parameters in a patient. In addition, we found talar position to be associated with hindfoot morphology, with the talus markedly more anterior in patients with cavus hindfoot morphology. In sum, these results help define normal ankle radiographic anatomy and the symmetry further indicates that such parameters on the uninjured contralateral side can be reliably used to aid in reduction of distal tibia and pilon fractures. For example, consider the treatment of a high-energy pilon fracture. Commonly, the talus translates (subluxates or dislocates) anteriorly or posteriorly with the injury energy. Many times, the plafond fragments are displaced and there is metaphyseal comminution and marginal impaction of the articular surface on individual fragments. In these situations, reduction assessment of the plafond surface can be difficult because there may not be specific fracture reads to use. The correct location of the talus in space compared with the contralateral side can serve as one confirmatory reduction read for the plafond shape and alignment. Future investigation is needed to determine the efficacy of contralateral imaging in assisting with fracture reduction.

This study has several limitations. First, because this was a retrospective study, we were not able to control for variation in technique quality on radiographs (ie, patient positioning, beam angle). In addition, we acknowledge that determination of the proximal diaphyseal axis may have been affected by the use of lateral weight-bearing ankle radiographs rather than full-length lateral tibia radiographs. If the object of interest is not centered in the radiograph beam, it can appear distorted because of the “wag” of the beam.^14^ This might cause the CORA angle to measure differently on a full-length radiograph centered at the central tibia compared with a radiograph centered at the ankle. However, we expect this to have a lesser effect in this study because all measurements were obtained using the same protocol. The determination of the proximal diaphyseal axis, as described in the *Methods*, provided satisfactory visualization of the tibial diaphysis and was a similar strategy used to determine a CORA in deformity analysis and other similar studies.^15^ Relatedly, interobserver reliability for CORA location was only “acceptable” (ICC = 0.73). Although not entirely unexpected, as this parameter was the most sensitive to user placement of the proximal diaphyseal and distal tibia axes, it still represents a limitation of this radiographic analysis.

Furthermore, we found that the DTAPA negatively correlated with patient age, demonstrating a lesser magnitude apex posterior angulation in older patients. These results suggest that osseous morphology may change over a lifetime, which is not a novel proposition and well-documented in the femur and spine.^16,17,18,19,20,21^ However, the correlation was small (r = 0.279, R2 = 0.078, *P* < 0.001) and limits clinical extrapolation of this finding. Additional analysis is required to confirm whether tibia morphology changes with age. Finally, we acknowledge that two-dimensional imaging cannot fully capture the complexities of three-dimensional hindfoot anatomy and motion and that an apparently anterior or posterior talus on lateral imaging may be partially related to flexion/extension or rotation of the talus. However, in fracture surgery, two-dimensional fluoroscopic imaging is typically the only continuously available imaging and the standard of care. If the location of the talus is restored after reduction on two-dimensional imaging, this is one clue for the surgeon to use to help determine whether the three-dimensional relationships and anatomy have been restored.

In conclusion, the distal tibia has a consistent apex posterior bow that is symmetric from side to side, but variable from patient to patient. Distal tibia osseous morphology is influenced by sex and age. Hindfoot alignment is related to distal tibia osseous morphology, with increased apex posterior angulation associated with planus hindfoot alignment. Cavus hindfoot alignment is associated with a more anterior position of the talus relative to the distal tibia axis. When treating distal tibia fractures surgically, restoration of sagittal plane alignment can be challenging. Knowledge that this apex posterior angulation exists, and is variable depending on hindfoot alignment, can be very helpful in interpreting intraoperative findings and restoring native anatomy. Assessment of contralateral limb anatomy, including foot morphology, may serve as one additional tool that the careful fracture surgeon can use to help understand and restore native anatomy.
